# Integrated transcriptome and metabolomics analyses revealed key functional genes in *Canna indica* under Cr stress

**DOI:** 10.1038/s41598-024-64877-w

**Published:** 2024-06-18

**Authors:** Sixi Zhu, Wei Zhao, Luying Sheng, Xiuqin Yang, Huan Mao, Suxia Sun, Zhongbing Chen

**Affiliations:** 1 College of Eco-Environment Engineering, The Karst Environmental Geological Hazard Prevention of Key Laboratory of State Ethnic Affairs Commission, Guizhou Minzu University, Guiyang, 550025 China; 2 Department of Applied Ecology, Faculty of Environmental Sciences, Czech University of Life Sciences Prague, Kamýcka 129, Praha-Suchdol, 16500 Czech Republic

**Keywords:** Chromium, *Canna indica*, Physiology, Transcriptome, Metabolome, Plant stress responses, Wetlands ecology

## Abstract

Chromium (Cr) can interfere with plant gene expression, change the content of metabolites and affect plant growth. However, the molecular response mechanism of wetland plants at different time sequences under Cr stress has yet to be fully understood. In this study, Canna indica was exposed to 100 mg/kg Cr-contaminated soil for 0, 7, 14, and 21 days and analyzed using untargeted metabolomics (LC–MS) and transcriptomics. The results showed that Cr stress increased the activities of superoxide dismutase (SOD), ascorbate peroxidase (APX) and peroxidase (POD), the contents of glutathione (GSH), malondialdehyde (MDA), and oxygen free radical (ROS), and inhibited the biosynthesis of photosynthetic pigments, thus leading to changes in plant growth and biomass. Metabonomics analysis showed that Cr stress mainly affected 12 metabolic pathways, involving 38 differentially expressed metabolites, including amino acids, phenylpropane, and flavonoids. By transcriptome analysis, a total of 16,247 differentially expressed genes (DEGs, 7710 up-regulated genes, and 8537 down-regulated genes) were identified, among which, at the early stage of stress (Cr contaminate seven days), *C. indica* responds to Cr toxicity mainly through galactose, starch and sucrose metabolism. With the extension of stress time, plant hormone signal transduction and MAPK signaling pathway in *C. indica* in the Cr14 (Cr contaminate 14 days) treatment group were significantly affected. Finally, in the late stage of stress (Cr21), *C. indica* co-defuses Cr toxicity by activating its Glutathione metabolism and Phenylpropanoid biosynthesis. In conclusion, this study revealed the molecular response mechanism of *C. indica* to Cr stress at different times through multi-omics methods.

## Introduction

Heavy metal pollution has become a global environmental problem^1^. In recent years, due to the influence of human activities, Cr has been widely distributed in soil and water^2^. Among them, industrial activities (such as electroplating, smelting, and mining) and agricultural activities (such as pesticide use and fertilizer application) are the primary sources of Cr pollution^3^. In nature, Cr exists in trivalent (Cr^3+^) and hexavalent (Cr^6+^) forms, with Cr^6+^ having good mobility and toxicity^4^. Although Cr is a non-essential element in plants, it can still accumulate in large amounts in plant roots and aboveground parts^5^. Even trace levels can harm plants' morphological, physiological, and molecular characteristics^3^. In addition, at high concentrations, Cr can lead to a variety of toxic symptoms in plants, such as inducing oxidative stress, resulting in excessive production of reactive oxygen species (ROS), reducing the activity of antioxidant enzymes, blocking the synthesis of photosynthetic pigments, inhibiting photosynthesis, thus affecting plant growth and development and reducing their biomass^6^. In addition, Cr is a non-biodegradable heavy metal element that can exist in plants for a long time and potentially threaten human and animal health through its spread through the food chain^7^. Therefore, the remediation of Cr-contaminated soil is necessary and urgent.

In the past few decades, several remediation methods for Cr contamination have emerged, among which physical, chemical, and biological methods have been successfully applied to the remediation of Cr-contaminated soils^8^. Compared with traditional restoration techniques, phytoremediation is an aesthetic, economical, and publicly recognized in-situ bioremediation technology^9^. It can provide an effective solution for soil removal, transfer, degradation, and fixation of Cr^10^. However, some challenges remain in its application, as phytoremediation mainly depends on the concentration of Cr in the soil^11^. Therefore, screening Cr-tolerant plants and understanding the molecular response mechanism of these plants on Cr tolerance is the focus of phytoremediation research, which will further promote the remediation effect of Cr-contaminated soil^12^. At present, some Cr-tolerant plants have been identified by studies. There are *Leersia hexandra*^13^, *C. indica*^14^, *Cyperus alternifolius*^15^, *Medicago sativa*^16^. These plants have evolved various defense and detoxification mechanisms to cope with heavy metal chromium (HMC) stress. The cell wall is the first physical barrier that effectively inhibits Cr from entering root cells^17,18^, which can significantly reduce Cr absorption and fix Cr in the cell wall. When the first barrier is breached, plants activate their antioxidant defense system and use vacuoles for compartments, thus alleviating the toxic effects of Cr^19^.

*C. indica* belongs to Canna indica, a perennial herbaceous species, showing a developed root system, strong adaptability to the living environment, rapid growth, large leaf area and other characteristics, and strong enrichment ability to heavy metals^20,21^. In treating contaminated wastewater containing heavy metals such as Cr, it is found that it has a robust, comprehensive tolerance, which can quickly adjust its own physiological and biochemical characteristics, showing strong tolerance^22^. At present, a large number of studies have focused on the response mechanism of *C. indica* to Cr stress in morphology, physiology, and biochemistry, including plant biomass, plant chelate (PC) synthesis, photosynthetic pigment, antioxidant defense system, and organic acid secretion^21,23,24^. Our previous studies showed that the contents of chlorophyll, malondialdehyde (MDA), and reduced glutamate (GSH) in *C. indica* seedlings changed significantly with increased Cr concentration. Moreover, the activities of enzymes related to the antioxidant mechanism (SOD, CAT, POD, and APX) were also changed^14^. However, the potential molecular mechanism underlying the response of *C. indica* to Cr stress remains largely unknown. In recent years, with the development of transcriptome sequencing (RNA-Seq) and metabolomics techniques, they have been widely used to reveal the different response mechanisms of different plants to Cr stress, including *Zea mays*^3^, *Helianthus annuus*^25^, *Arabidopsis thaliana*^26^, *Sorghum bicolor*^27^. Therefore, taking *C. indica* as the research object and combining multi-omics techniques, the knowledge gap of *C. indica'*s response to Cr stress can be eliminated at the molecular level.

Therefore, physiological, transcriptomic, and metabolomic methods were used in this study to analyze the molecular response mechanism of *C. indica* to Cr stress at different exposure times. We hypothesized that Cr stress could induce the abnormal expression of many genes and metabolites related to the antioxidant and detoxification mechanisms of *C. indica*. The purpose of this study was to: (a) analyze the accumulation and transport of Cr by *C. indica* and its physiological changes under Cr stress at different exposure times; (b) identify the key metabolic pathways based on differentially expressed genes (DEGs) and metabolites (DEMs); (c) reveal the molecular mechanism of *C. indica* tolerance under Cr stress at different exposure times, to provide a theoretical basis for phytoremediation of soil Cr pollution, and identify essential phytoremediation candidate genes to provide a theoretical basis for future research.

## Materials and methods

### Plant cultures and Cr treatment

The background value of heavy metal Cr in Guizhou Province is 95.9 mg kg^−1^^15,28–30^. The *C. indica* seedlings used in this study were hydroponically grown by Songnan Plant Seedling Company of Luzhi town, Suzhou City. Firstly, *C. indica* seeds of the same size were screened. After sterilization (The seeds were soaked in 1% sodium hypochlorite solution for 10–30 min and repeatedly cleaned in deionized water before being placed in Petri dishes), seeds were cultured in Petri dishes, waiting for germination, and then moved to nutrient water for hydroponics. When the plants grew to about 10 cm, we purchased seedlings from the company and selected seedlings with similar growth conditions for the experiment. The selected *C. indica* seedlings were surface disinfected with 75% ethanol and 1% sodium hypochlorite solution for 10 s and 15 min. Carefully washed with deionized water five times, furthermore transplanted in the greenhouse (500 g of soil per pot). All seedlings were then earth culture in a controlled greenhouse (176 μmol m^2^ s^−1^ light intensity, 12 h photoperiod, 25 °C constant temperature). To ensure the accuracy of the controlled test, we purchased the culture black soil that was not contaminated with Cr from this company. Seedlings were domesticated in the greenhouse for 30 days before exposure to Cr. Hoagland solution (15 mL; Table S11) and deionized water (15 mL) were added to the pot every 15 days and every three days, respectively, and Hoagland nutrient solution was not added after Cr stress. After domestication, *C. indica* seedlings were treated with Cr stress. Sixty-three seedlings with similar growth conditions were randomly divided into seven groups with nine plants in each group (three plants per pot): four groups were the control group (0 mg/kg K_2_Cr_2_O_7_), and the other three groups were the Cr treatment group (100 mg/kg K_2_Cr_2_O_7_; Configure K_2_Cr_2_O_7_ solution). Seedlings were treated at 0, 7, 14, and 21 days of Cr stress (nine plants at 0 days, eighteen plants at 7 days, eighteen plants at 14 days, and eighteen plants at 21 days). Prepare the solution of 100 mg/kg K_2_Cr_2_O_7_ and pour it around the plant (100 mL volumetric bottle configuration, add once, add 50 mL). Root (R) tissue from both Cr-treated and untreated seedlings was sampled simultaneously on corresponding days and immediately frozen in liquid nitrogen and stored in a − 80 °C freezer until further treatment. Finally, 21 groups of samples were collected for transcriptomic and metabolomic analysis. Similarly, 21 sets of samples were collected in this way for biochemical analysis (collect 3 plant roots into a group). This study have permission to collect *C. indica* plant. And all methods were carried out in accordance with relevant guidelines in the method section.

### Cr content in soil and plants

Rhizosphere soil samples and leaf samples were digested in a 1:3 v/v mixture of HCL and HNO_3_. The mixture was then added to a 50 ml volumetric flask and diluted with deionized water. To test the cadmium content, the Cd content of experimental samples was measured using ICP-MS^30^.

### Soil indicator and physiological index of plants

#### Soil physicochemical properties

Soil pH and conductivity were determined using a potentiometric method using STARTER 2100 and LEI-CI DDS-307A. Soil organic matter (SOM) was measured by K_2_Cr_2_O_7_-H_2_SO_4_ oxidation-external heating method^31,32^.

#### Analysis of plant physiological and biochemical indicators

Chlorophyll and carotenoids are insoluble in water and soluble in organic solvents. Chlorophylls and carotenoids were extracted crudely from organic solvents, and chlorophyll a, b, and carotenoids had the maximum absorption at 645 nm, 663 nm, and 470 nm. DTNB: 5,5'-dithio-bis- (2-nitrobenzoic acid), which can react with glutathione (GSH) to produce 2-nitro- 5-mercaptobenzoic acid and glutathione disulfide (GSSG). Since 2-nitro-5-mercaptobenzoic acid is a yellow product, the amount of glutathione in the sample can be determined by measuring its maximum absorption at 412 nm. Under the action of sulfuric acid, sugars are dehydrated to form furfural or hydroxymethylfurfural, and anthrone and furfural or hydroxymethylfurfural are dehydrated and condensed to form a blue-green derivative, which has a characteristic absorption peak at 620 nm, and the change of the absorbance value can be used to detect the content of soluble sugars in plants quantitatively. Malondialdehyde (MDA) can be condensed with thiobarbituric acid (TBA) under acidic and high-temperature conditions to produce the brownish-red color trimethoprim (3,5,5-trimethyloxazole-2,4-dione), which has a maximum absorption wavelength of 532 nm. Intracellular reactive oxygen species can oxidize non-fluorescent DCFH to produce fluorescent DCF. Detecting the fluorescence of DCF tells us the level of intracellular reactive oxygen species. The peroxidase (POD) catalyzes the decomposition of hydrogen peroxide into water and oxygen, and the oxygen oxidizes pyrogallic gallic acid to form a yellow product. Oxygen oxidizes pyrogallic gallic acid to form a yellow product, and the enzyme activity was determined by measuring the absorbance change at 420 nm. The enzyme activity was determined by measuring the change in absorbance at 420 nm. Superoxide anion (O_2_^-^) is produced by xanthine and xanthine oxidase reaction system, and O^−^ reacts with WST-1 to produce water-soluble yellow filth, which is absorbed at 450 nm.

The content of chlorophyll, carotenoid, glutathione (GSH), soluble sugar, malondialdehyde (MDA), and reactive oxygen species (ROS) was detected using Suzhou Keming Biotechnology Co., LTD (www.cominbio.com) standard kit. The activities of peroxidase (POD), superoxide dismutase (SOD), and ascorbate peroxidase (APX) were measured using Suzhou Keming Biotechnology Co., LTD (www.cominbio.com) standard kits.

### Transcriptome analysis

RNA-sequencing (RNA-Seq) was applied to determine the changes in gene expression in the roots of *C. indica* under Cr stress at different times. Root tissue (3 replicates per group) of 0.5 g was taken from Cr-treated and untreated *C. indica* seedlings at days 0, 7, 14, and 21 and were frozen in liquid nitrogen. The RNA of root samples of *C. indica* under CK and Cr treatments (including three biological replications) was extracted by TRIzol^®^ Reagent (Invitrogen, USA), purified by Plant RNA Purification Reagent (Invitrogen company), and sequenced on the HiSeq 6000 Illumina sequencing platform by Shanghai Majorbio Bio-pharm Technology Co. Ltd, China^33,34^.

### Metabolomics analysis

Root tissue of 0, 7, 14, and 21-day time series from Cr-treated and untreated *C. indica* seedlings (3 replicates per group) was taken at about 1 g (3 replicates per group), weighed, and frozen in liquid nitrogen. After natural air drying, root samples were ground to powder, and metabolites were extracted and analyzed. The 60 mg ground powder was ultrasonically extracted with 0.6 mL methanol/water (7:3, v/v) for 30 min followed by 20 min incubation at − 20 °C and an internal standard of L-2-Cl-Phe (0.3 mg mL^−1^). The extracts were centrifuged at 14,000 *rpm* for 10 min at 4 °C. Then, 200 μl of supernatant was filtered through a 0.2 μm filter and measured using a Waters VION IMS Q-TOF Mass Spectrometer equipped with an electrospray interface (Waters Corporation, Milford, MA, USA) platform as described elsewhere^35^.

### Expression analysis using quantitative RT-PCR

Total RNA (5 µg) isolated from *C. indica* exposed to different treatments viz., Cr0, Cr7, Cr14 and Cr21 was reverse transcribed by using SuperScriptII (Fermentas, USA). The synthesized cDNA was diluted in DEPC water in the ratio of 1:5 and subjected to quantitative RT-PCR analysis. Each qRT-PCR reaction was performed in a total reaction volume of 20 μl for each set of selected genes by using Fast SYBR Green PCR Master Mix (Agilent Technologies, USA). The qRT-PCR reactions were performed by using the following cycle conditions: an initial 94 °C for 2 min, followed by 30 cycles of 94 °C for 30 s, 60 °C for 30 s, and 72 °C for 30 s, and the final 5 min extension at 72 °C. After obtaining the ct-value for each reaction, the relative expression was calculated by 2^−delta Ct method.

### Statistical analysis

One-way ANOVA and IBM SPSS 26.0 (Chicago, USA) were used for statistical analysis, and the nonparametric Kruskal–Wallis test was used to analyze the data when the assumptions of normal distribution and homogeneity of variance were not met. Charts were drawn using Origin 2021 and Adobe Illustrator. Visualization of transcriptome and metabolome data was performed using the online platform (www.majorbio.com).

## Results

### Physiological changes and Cr accumulation of *C. indica* under Cr stress

**Figure 1:**
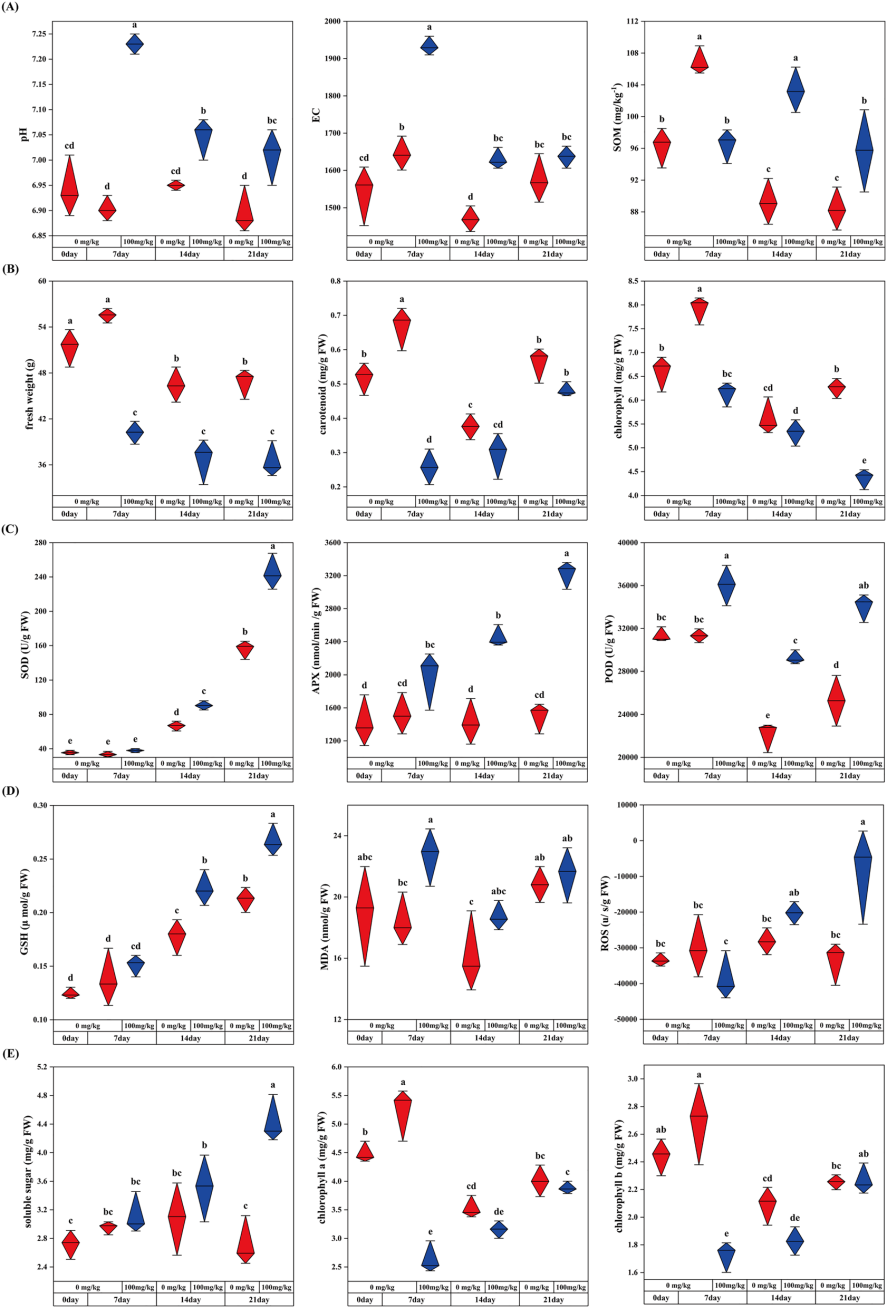
Effects of Cr stress on soil physicochemical properties (pH, EC, and SOM) and physiological and biochemical indexes of *C. indica* (Freshweight, Carotenoid, Chlorophyll, SOD, APX, POD, GSH, MDA, ROS, Soluble sugar, Chlorophyll a, and Chlorophyll b). Data within the same followed by a string of the same lowercase letters are not significantly different (P > 0.05). At the same time, a series of other letters show a significant difference (P < 0.05).

In this study, compared with group Cr0, pH and EC in soil increased significantly after Cr(VI) was added, but their values decreased substantially with increased stress time. Meanwhile, soil organic matter (SOM) content decreased significantly with increased stress time; however, it was significantly higher than the control group at 14 and 21 days (Fig. 1A). In addition, prolonged Cr stress time significantly decreased the biomass of *C. indica*. In addition, with the increased Cr stress time, the contents of carotenoid and total chlorophyll also reduced significantly, especially in group Cr7, which decreased by 50.22% and 33.85% compared with group Cr0 (Fig. 1B). Meanwhile, with the increase of Cr stress time, the contents of Cr(VI) and Cr(III) in soil showed a trend of first increasing and then decreasing. It was found by measuring the Cr content in the leaves of *C. indica* showed a trend of significantly increasing all the time and reached the maximum value in group Cr21 (Table 1).

**Table 1:** Accumulation and transport of heavy metal Cr by *Canna indica*.

Group	Cr(VI) in soil (mg/kg)	Cr(III) in soil (mg/kg)	Cr(VI) in leaves (mg/kg)	Cr(III) in leaves (mg/kg)
Cr0	12.40 ± 0.26d	29.36 ± 0.37f	0.44 ± 0.07d	1.04 ± 0.11c
CK7	13.27 ± 0.31b	37.89 ± 0.20d	0.39 ± 0.05d	1.11 ± 0.05c
Cr7	14.60 ± 0.26a	117.84 ± 2.05a	4.75 ± 0.58c	9.45 ± 0.41b
CK14	12.30 ± 0.36d	33.62 ± 0.17e	0.56 ± 0.05d	1.19 ± 0.04c
Cr14	13.10 ± 0.28bc	85.68 ± 1.32b	6.22 ± 0.82b	14.54 ± 0.46a
CK21	12.50 ± 0.37d	28.13 ± 0.91f	0.73 ± 0.06d	1.36 ± 0.15c
Cr21	12.67 ± 0.15cd	65.93 ± 0.96c	8.82 ± 0.45a	15.34 ± 1.73a

Data within the same followed by a string of the same lowercase letters are not significantly different (P > 0.05). At the same time, a string of different letters shows a significant difference (P < 0.05).

In this study, after the addition of Cr(VI), the activities of superoxide dismutase (SOD) and APX(ascorbate peroxidase) in C indica of group Cr21 were increased by 75.49% and 56% compared with that of group Cr0, respectively (Fig. 1C). In contrast, POD(peroxidase) activity showed the highest value in the Cr7 group, which increased by 13.02% compared with the Cr0 group. With increased Cr stress time, the activity of antioxidant enzymes showed an increasing trend. In addition, it can be seen from the change of glutathione (GSH) and soluble sugar content in *C. indica* that the content of GSH and soluble sugar is increasing (p < 0.05), and its maximum value was found in group Cr21. At the same time, the contents of malondialdehyde (MDA) and reactive oxygen species (ROS) were also significantly increased after Cr(VI) was added and reached their peaks in Cr7 and Cr21 groups, respectively (Fig. 1D,E).

### Metabolomic analysis

#### Metabolic changes of *C. indica* roots under Cr stress

**Figure 2:**
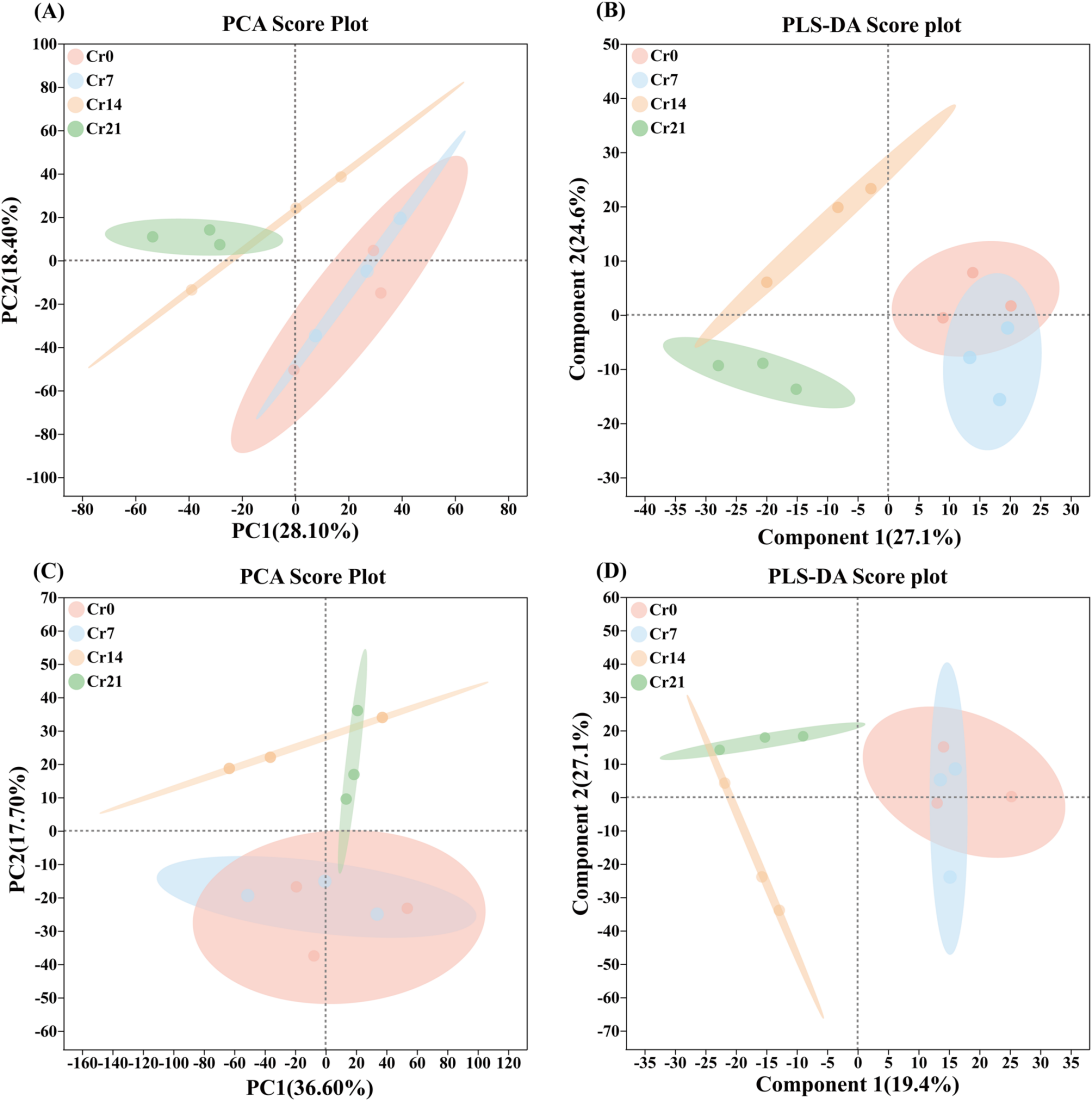
PCA and PLS-DA plot metabolic profiles in *C. indica* root among different groups in both the positive ion (**A**,**B**) and negative ion (**C**,**D**) modes under Cr treatments, respectively. The higher the similarity of species composition, the more the area of shaded part crosses. Shanghai Majorbio Bio-pharm Technology platform provides all KEGG materials, and the copyright license for image use is obtained.

In this study, non-targeted metabolomics (LC–MS) was used to study the metabolism of *C. indica* roots to identify the different metabolites associated with Cr immobilization in *C. indica* roots to understand better the stress response mechanism of *C. indica* roots under Cr stress. The results showed that Cr stress had little effect on the metabolites in the roots of *C. indica* under cationic mode (Fig. 2A). At the same time, there was a significant partitioning phenomenon between Cr14 and Cr21 groups and the Cr0 groups due to the significant differences between groups in the Cion mode (Fig. 2A,C). This study showed that the PLS-DA scatter in all treatment groups showed an apparent partitioning phenomenon in the cationic mode (Fig. 2B,D). Meanwhile, in the cationic mode, the differential interpretation rate of PLS-DA analysis reached 51.7%, reflecting this data's reliability and applicability for future studies (Fig. 2B). In summary, these results suggest that Cr stress can significantly affect the composition of metabolites in the roots of *C. indica*.

#### Cluster analysis of DEMs in the roots of *C. indica*

**Figure 3:**
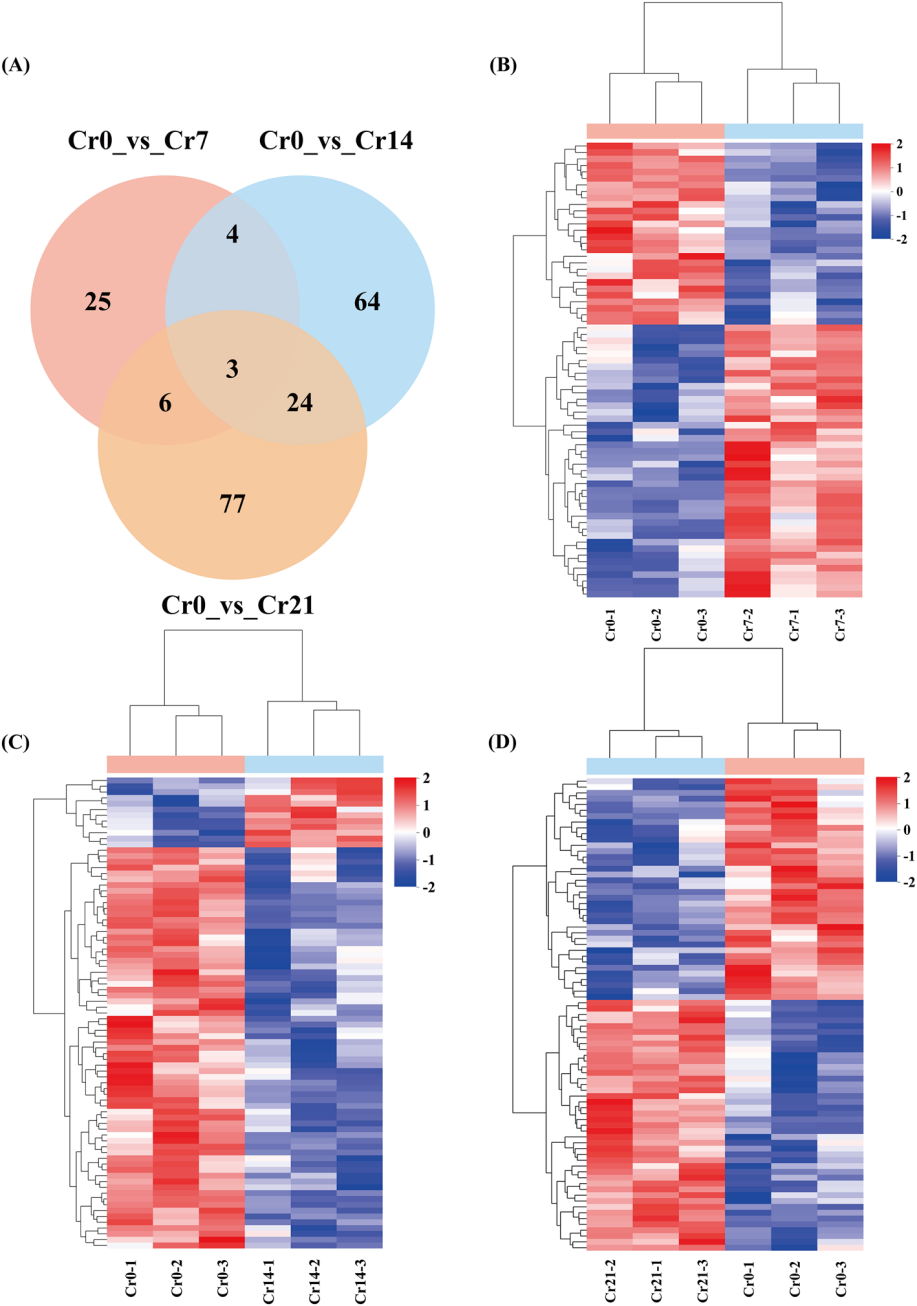
Quality control of Metabolomics data and Changes in DEM expression. (**A**) Compare the Veen graph of the number of DEMs in groups pairwise. The overlap represents the number of metabolites common to each comparison group, and the non-overlap represents the number of metabolites unique to the comparison group, (**B**–**D**) Heatmap showing the results of the clustering analysis of DEMs. The stronger the positive correlation the darker the red, and the stronger the negative correlation the darker the blue. Shanghai Majorbio Bio-pharm Technology platform provides all KEGG materials, and the copyright license for image use is obtained.

38 DEMs were detected in the Cr0 vs Cr7 comparison group, with 24 up-regulated and 14 down-regulated metabolites. Up-regulated metabolites include Chrysoidine free base and Citicoline. Similarly, 95 DEMs were detected in the Cr0 vs Cr14 comparison group (15 up-regulated and 80 down-regulated). Gln Val Tyr Asp, Phytosphingosine-1-P, Methyl jasmonate, M-Coumaric acid, and P-Tolualdehyde were particularly abundant in the Cr-contaminated group. As Cr stress duration increased, more up-regulated DEMs were detected in the Cr0 vs Cr21 comparison group. Asp Ile Gln Gly, Gln Val Tyr Asp, L-Aspartic acid, L-Glutamate, M-Coumaric acid, Mevalonic acid, and Flumiclorac-pentyl were most abundant (Fig. 3A–D; Table S1). Notably, Gentiopicrin was significantly expressed in all three comparison groups (Table S1). Meanwhile, we identified the top ten DEMs in each comparison group based on changes in differential metabolites (Fig. S2A). The volcano map visually illustrates DEM changes (Fig. S2B).

Under Cr stress, Among the secondary metabolite classes, the DEMs are mainly Flavonoids (28.57%), Phenylpropanoids (20%), Terpenoids (31.43%), Fatty acids-related compounds (8.57%), and Alkaloids (5.7%) (Fig. S1A; Table S2), in the classification of lipid compounds, It is mainly composed of Fatty acyls (37.5%), Glycerolipids (12.5%), Glycerophospholipids (36.54%), Polyketides (8.65%), and Sterol lipids (8.65%) (Fig. S1B; Table S2). These results suggest that amino acids, phenylpropanoids, flavonoids, terpenoids, Fatty acyls, and Glycerophospholipids may be crucial in detoxifying Cr in *C. indica* roots.

#### Analysis of enrichment of KEGG functional pathway in DEMs in *C. indica* roots

**Figure 4:**
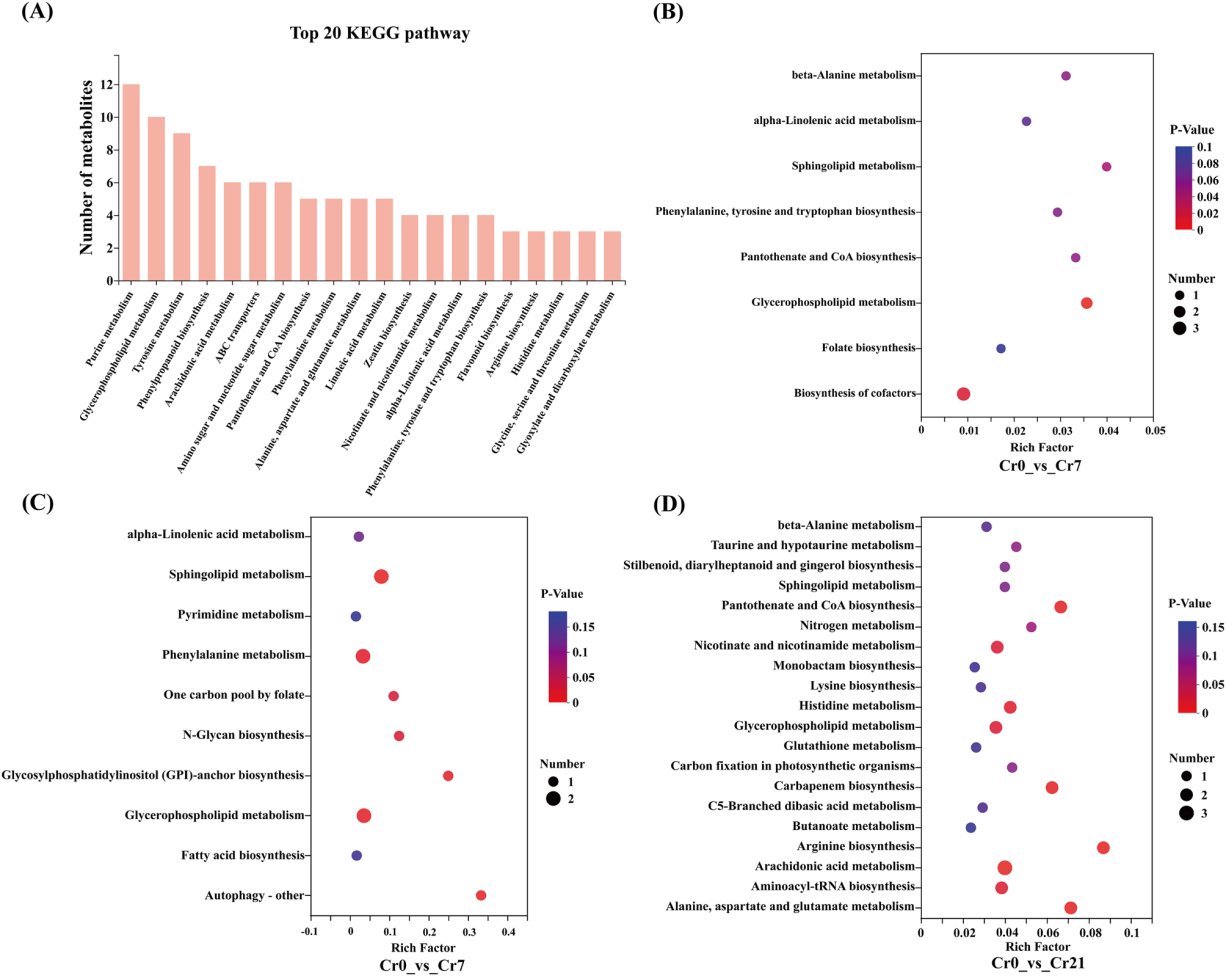
Enrichment analysis of KEGG functional pathways. (**A**) Top 20 functional pathways for metabolite enrichment. From left to right, the number of metabolites in the column was ranked from high to low. The higher the column, the more metabolites are involved in this pathway among the identified metabolites. (**B**–**D**) The top 20 pathways of the significance of the up-regulated and down-regulated DEMs on KEGG. The X-axis represented the rich factor, and the Y-axis represented the pathway's name. The bubble size represents the number of DEMs involved. The bubbles color indicates the enrichment degree of the path. Shanghai Majorbio Bio-pharm Technology platform provides all KEGG materials, and the copyright license for image use is obtained.

Through the enrichment analysis of the KEGG pathway, the biological pathways between different comparison groups were determined to further explore the metabolic mechanism of *C. indica* to Cr stress. First, we searched and annotated the DEMs in *C. indica* roots under different Cr treatments and screened out the top 20 metabolic pathways in enrichment (Fig. 4A; Table S3). The Biosynthesis of cofactors was mainly enriched in the Cr0 vs Cr7 comparison group. Phenylalanine metabolism, Sphingolipid, N-Glycan, Glycosylphosphatidylinositol (GPI)-anchor biosynthesis, and Autophagy was enriched primarily on the Cr0 vs Cr14 comparison group. With prolonged stress, The significantly enriched metabolic pathways in the Cr0 vs Cr21 comparison group mainly included Histidine, Arachidonic acid, Alanine, aspartate and glutamate, Nicotinate, nicotinamide metabolism, and Arginine biosynthesis. It should be noted that Glycerophospholipid metabolism is significantly enriched in Cr7, Cr14, and Cr21 (Fig. 4B–D; Table S4). Therefore, these DEMs-enriched pathways in *C. indica* may play an essential role in plant response to Cr stress.

### Transcriptomic analysis

#### Gene function annotation

Novaseq 6000 (Illumina) was used for transcriptional sequencing to understand gene expression changes in *C. indica* roots under Cr stress. In this study, 216,527 genes and 516,953 transcripts were identified in 6 different databases, among which the NR database had the highest annotation rate, and 49,065 genes were significantly annotated (Table S5; Fig. S3). In addition, a total of 155.92 Gb of Clean Data was obtained from 21 samples in this study, and all Q20 and Q30 values were greater than 98% and 94%, respectively (Table S6). These results reflect the reliability and applicability of this data for future studies.

#### Analysis of differentially expressed genes (DEGs) and changes in gene expression

**Figure 5:**
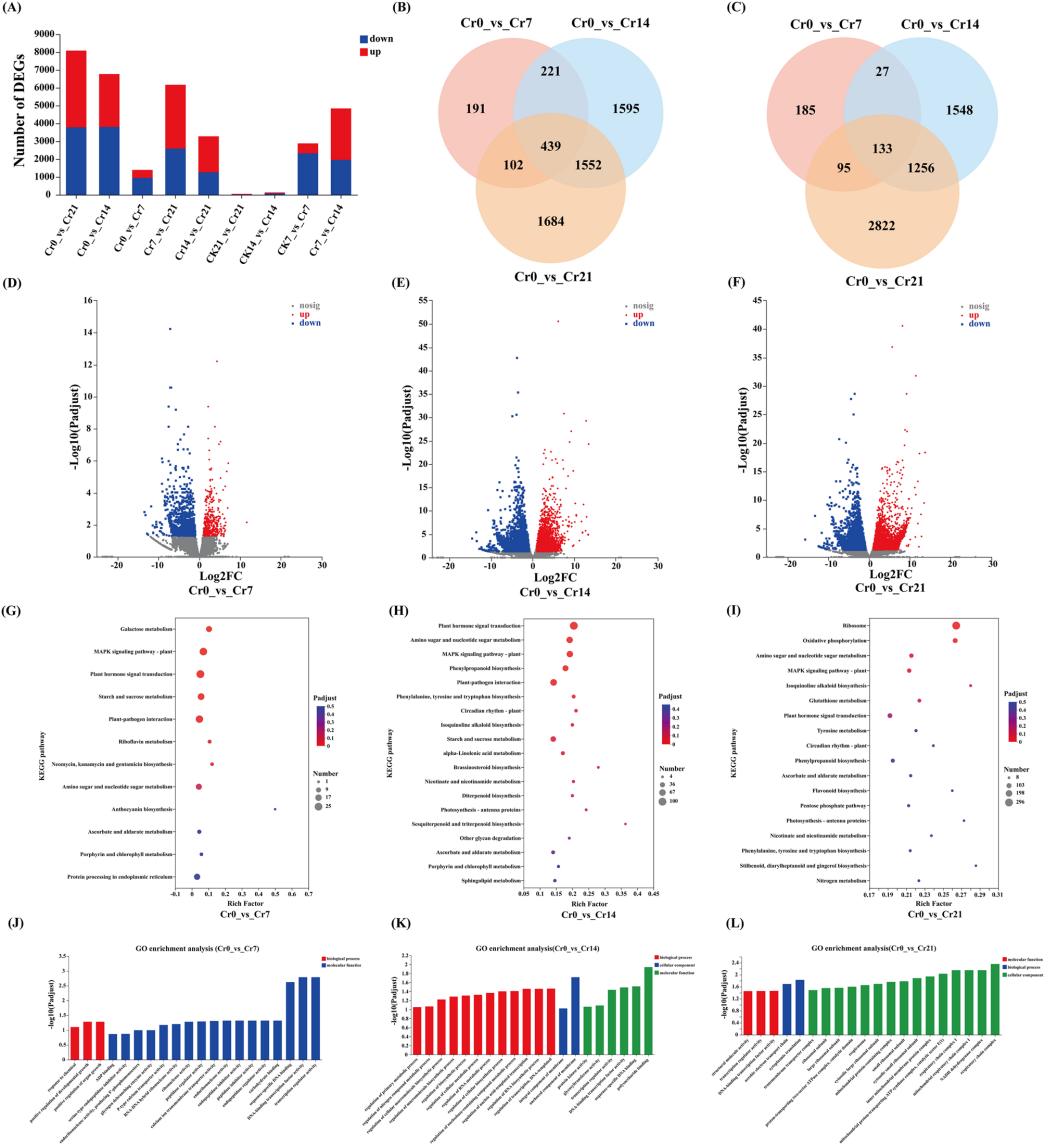
Changes in DEG expression, (**A**) Up-regulation and down-regulation of DEGs, red represents up-regulation, blue represents down-regulation. (**B**,**C**)Veen plot of pairwise comparison of the group's number of up-regulation and down-regulation DEGs. The overlap means the number of metabolites common to each comparison group, and the non-overlap represents the number of metabolites unique to the comparison group. (**D**–**F**) Volcano plots of DEGs up-regulation and down-regulation (**G**–**I**) The top 20 pathways of the significance of the up-regulated and down-regulated DEGs on KEGG. The X-axis represented the rich factor, and the Y-axis represented the pathway's name. The bubble size represents the number of DEGs involved. The bubbles color indicates the enrichment degree of the path (**J**–**L**) GO pathway enrichment analysis. Shanghai Majorbio Bio-pharm Technology platform provides all KEGG materials, and the copyright license for image use is obtained.

This study compared the up-regulated and down-regulated DEGs in the Cr0 group with those in the Cr7, Cr14, and Cr21 treatment groups. The results showed that with the extension of Cr stress time, the up-regulated genes of *C. indica* root increased significantly, with 1393(440 up-regulated and 953 down-regulated) in the three comparison groups, respectively. 6771(2964 up-regulated and 3807 down-regulated) and 8083(4306 up-regulated and 3777 down-regulated) (Fig. 5A). Among them, the top 15 up-regulated and down-regulated genes with differentially expressed levels among the comparison groups are shown in Table S7. Meanwhile, the total number of up-regulated and down-regulated DEGs among the comparison groups was 439 and 133, respectively (Fig. 5B,C). In addition, based on the magnitude and significance of the observed stress effects, we further evaluated the overall gene expression between the comparison groups using volcanic maps. Using volcanic maps, we evaluated DEGs in the three comparison groups (Fig. 5D–F).

#### KEGG and GO functional enrichment analysis of DEGs

In the Cr0 vs Cr7 comparison group, DEGs were significantly enriched in DNA-binding transcription factor and regulator activity. Participate in the regulation of RNA biosynthetic process, regulation of transcription, DNA-templated, and an anchored component of membrane DEGs were mainly significantly enriched in the Cr0 vs Cr14 comparison group. With the extension of Cr stress time, The functional pathways significantly enriched in the Cr0 vs Cr21 comparison group mainly include aerobic electron transport chain, cytoplasmic translation, inner mitochondrial membrane protein complex, NADH dehydrogenase complex, and respiratory chain complex (Fig. 5J–L; Tables S8, S9).

In the Cr0 vs Cr7 comparison group, upregulated DEGs (TRINITY_DN124_c0_g2) are mainly involved in galactose metabolism, and the DEGs (TRINITY_DN14773_c0_g1, TRINITY_DN15854_c0_g2, TRINITY_DN16130_c0_g3) involved in Phenylpropanoid biosynthesis and Starch and sucrose metabolism are primarily enriched in the Cr0 vs Cr14 comparison group. With the extension of Cr stress time, The significantly enriched functional pathways in the Cr0 vs Cr21 comparison group mainly included Ribosome, Oxidative phosphorylation, Glutathione metabolism DEGs (TRINITY_DN3914_c0_g1, TRINITY_DN52144_c0_g1, TRINITY_DN690_c0_g1), and Isoquinoline alkaloid biosynthesis. It is worth noting that starch and sucrose metabolism, MAPK signaling pathway-plant DEGs (TRINITY_DN1298_c0_g1, TRINITY_DN11035_c0_g1), and Plant hormone signal transduction DEGs (TRINITY_DN1298_c1_g1, TRINITY_DN12863_c0_g1, TRINITY_DN12505_c0_g1) were significantly enriched in Cr7 and Cr14. However, with the extension of Cr stress time, the enrichment degree of these three functional pathways decreased significantly (Fig. 5G–I; Table S10).

#### Analysis of co-expression network of transcription factors and weighted genes

**Figure 6:**
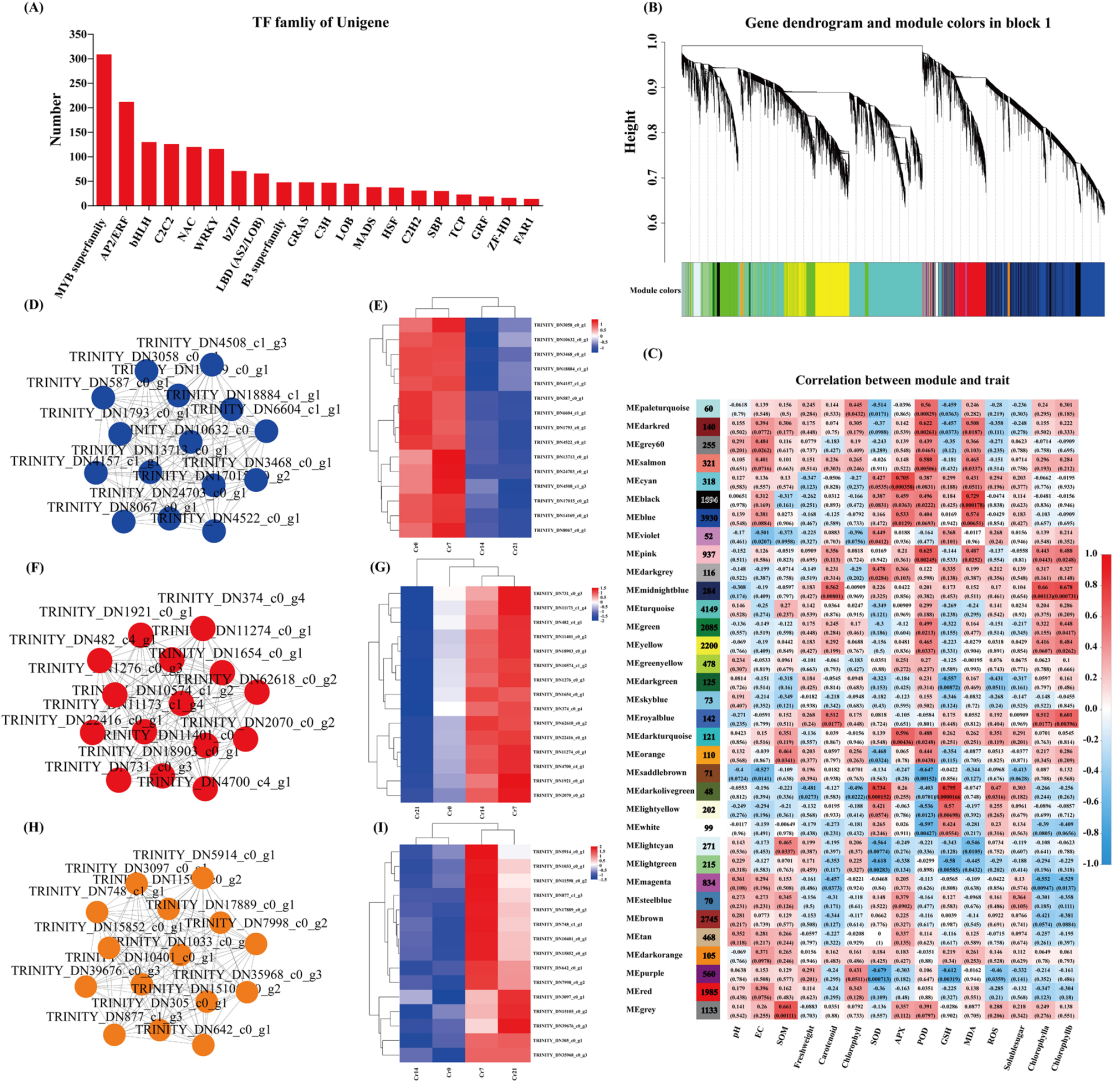
Results of TF and WGCNA. (**A**) The number of top 20 TFs. (**B**) Hierarchical clustering tree showing coexpression modules identified by WGCNA. (**C**) Module sample association relationships. (**D**,**E**) midnight blue module central gene symbiosis network map and clustering heat map, (**F**,**G**) black module major gene symbiosis network and clustering heat map, (**H**,**I**) Symbiotic network map and clustering heat map of dark olive-green module center gene. Shanghai Majorbio Bio-pharm Technology platform provides all KEGG materials, and the copyright license for image use is obtained.

In this study, a total of 1619 transcription factors (TFs) were identified from 33 transcription factor families. The top 5 families were MYB_superfamily, AP2/ERF, bHLH, C2C2, and NAC (Fig. 6A). Weighted gene co-expression network analysis (WGCNA) was used to investigate the relationship between genes and physiology and biochemistry in *C. indica*. Thirty-four gene modules were identified, including 26,296 genes (Fig. 6B,C). Among them, the central gene of the MEmidnightblue module was positively correlated with the content of photosynthetic pigments (P < 0.001), and through the network diagram and correlation heat map, it can be seen that DEGs regulating photosynthetic pigments were significantly up-regulated at the early stage of stress, but significantly down-regulated at the late stage of stress (Fig. 6D,E). The central gene of the MEblack module was positively correlated with the degree of lipid peroxidation (MDA) in plants (P < 0.001). According to the screened central genes, lipid peroxidation in Cr7 and Cr14 groups was significantly enhanced but significantly weakened at the later stage of stress (Fig. 6F,G). The central gene of the MEdarkolivegreen module was positively correlated with the activity of antioxidant enzymes (SOD) and the content of non-enzymatic antioxidant substances (GSH) in plants (P < 0.001), whose central gene was significantly enhanced in Cr7 and Cr21 groups (Fig. 6H,I and Table S12).

#### Validation of the DEGs results by qRT-PCR analysis

**Figure 7:**
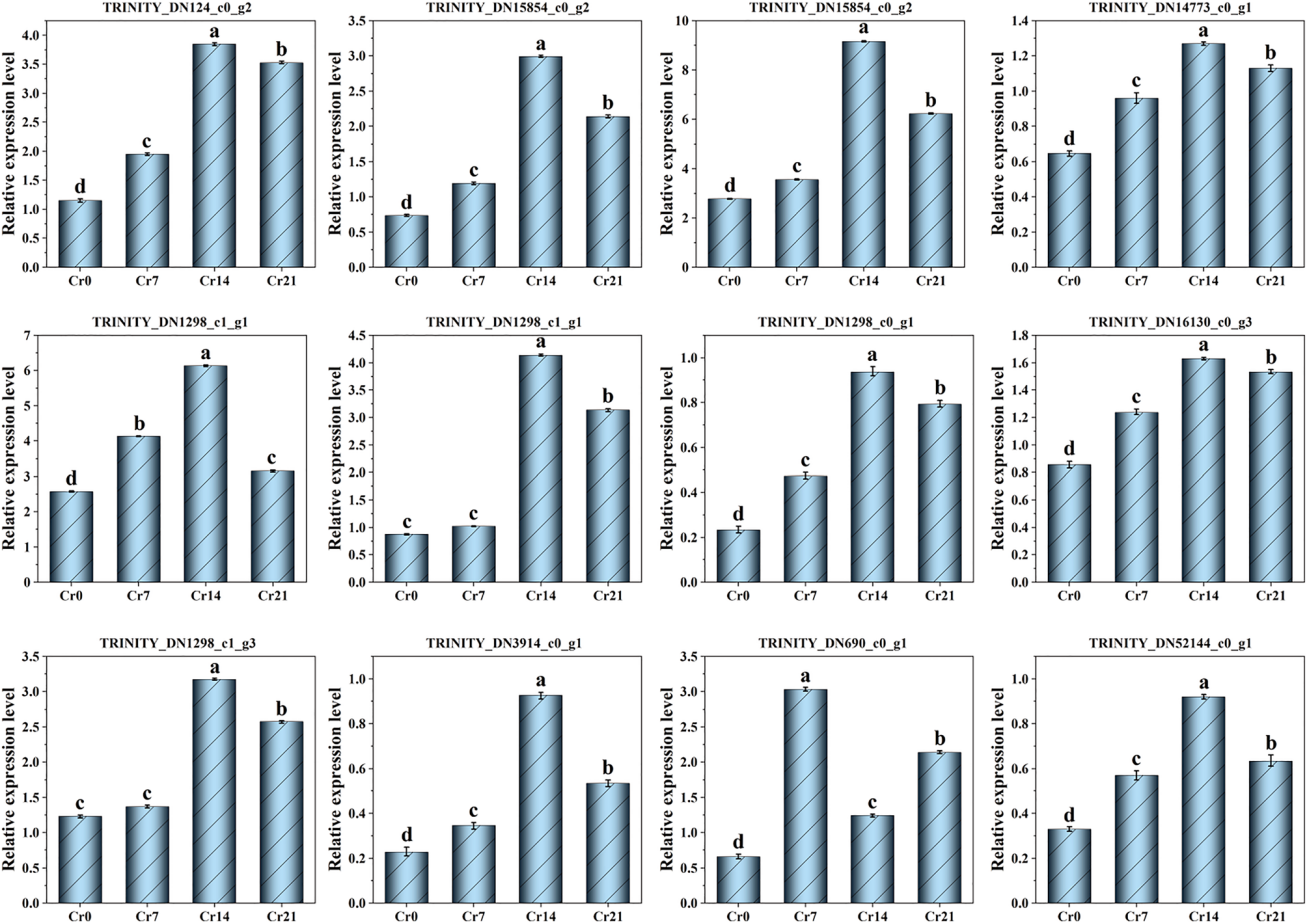
Results of the quantitative PCR (qRT-PCR) results for the mRNA expression profiles of differentially expressed genes (DEGs). The significance of differences in samples was performed by combining one-way ANOVAs and Tukey’s test. The significant difference was 0.01 (P < 0.01). Data within the same followed by a string of the same lowercase letters are not significantly different (P > 0.05). At the same time, a string of different letters shows a significant difference (P < 0.05). The detailed primer information is shown in Table S13. Galactose metabolism DEGs (TRINITY_DN124_c0_g2), Starch and sucrose metabolism DEGs (TRINITY_DN14773_c0_g1, TRINITY_DN15854_c0_g2, TRINITY_DN16130_c0_g3), Glutathione metabolism DEGs (TRINITY_DN3914_c0_g1, TRINITY_DN52144_c0_g1, TRINITY_DN690_c0_g1) 、MAPK signaling pathway-plant DEGs (TRINITY_DN1298_c0_g1, TRINITY_DN11035_c0_g1) and Plant hormone signal transduction DEGs (TRINITY_DN1298_c1_g1, TRINITY_DN12863_c0_g1, TRINITY_DN12505_c0_g1).

To verify the reliability of the RNA-Seq data, 12 DEGs were selected and validated by qRT-PCR. Selected DEGs (Galactose metabolism DEGs (TRINITY_DN124_c0_g2), Starch and sucrose metabolism DEGs (TRINITY_DN14773_c0_g1, TRINITY_DN15854_c0_g2, TRINITY_DN16130_c0_g3), Glutathione metabolism DEGs (TRINITY_DN3914_c0_g1, TRINITY_DN52144_c0_g1), MAPK signaling pathway-plant DEGs (TRINITY_DN1298_c0_g1, TRINITY_DN11035_c0_g1) and Plant hormone signal transduction DEGs (TRINITY_DN1298_c1_g1, TRINITY_DN12863_c0_g1, TRINITY_DN12505_c0_g1)) the trend was consistent with Illumina sequencing. Only the TRINITY_DN690_c0_g1 gene showed a similar expression trend that was not consistent with Illumina sequencing, with an expression trend agreement rate of 91.7%, indicating that the RNA-Seq analysis data results were reliable (Fig. 7).

## Discussion

### Physiological response changes of *C. indica* under Cr stress

Cr is almost not involved in any metabolic pathways in plants, but its toxicity will hinder and affect plant growth and development's physiological and biochemical processes^36^. Among them, changes in plant physiological characteristics are usually related to homeostasis and stress strategies^37^. Plants can enhance their tolerance to heavy metals through their antioxidant mechanisms, energy metabolism, and hormone transduction processes^38^. This study showed that after *C. indica* was exposed to Cr stress, leaves' carotenoid, and chlorophyll contents were significantly reduced, and plant growth was significantly inhibited (Fig. 1B,E). This is in contrast to previous studies in *Solanum lycopersicum L*.^11^, *rice*^5^, and *cauliflower*^4^, indicating that plant photosynthesis may be interfered with by Cr stress. Meanwhile, in this study, the central gene screened by WGCNA in the MEmidnightblue module was significantly positively correlated with the content of photosynthetic pigments. However, its gene expression was significantly down-regulated with the extension of stress time (Fig. 6D,E), which may explain why photosynthetic pigments decreased during stress. Plants can activate endogenous defense mechanisms in response to ROS-induced oxidative stress. The defense system mainly comprises antioxidant enzymes and chelates, such as SOD, APX, and POD, which can scavenge free radicals and neutralize intermediates with oxidative toxicity to maintain plants' homeostasis, thus reducing oxidative damage in plant cells^39^. El Rasafi et al.^40^ pointed out in a review study on plant response to heavy metal stress that plants reduce membrane lipid peroxidation and accumulation of intracellular reactive oxygen species mainly by increasing the activities of antioxidant enzymes (POD, SOD, and APX), to maintain their normal metabolic activities. This study observed that the activity of antioxidant enzymes (SOD, APX, and POD) and the contents of GSH and soluble sugar increased significantly with the extension of Cr stress time, consistent with previous studies results. Among them, soluble sugar could serve as energy storage substances for plants, signal transduction, and osmotic regulation substances, playing a pivotal role in plant growth and development and stress response^28^. GSH is a widely recognized essential plant metabolite and is an antioxidant and detoxifier, a precursor to phytochelatin. It can bind with Cr, Pb, and other heavy metals, significantly reduce its mobility and bioavailability, and greatly enhance plant tolerance to heavy metals^36,41,42^. It should be noted that the oxidative stress system of *C. indica* does not activate to the highest level at the initial stage of stress but gradually strengthens with the extension of stress time.

### Transcriptional metabolic response of *C. indica* to Cr stress under time series

**Figure 8:**
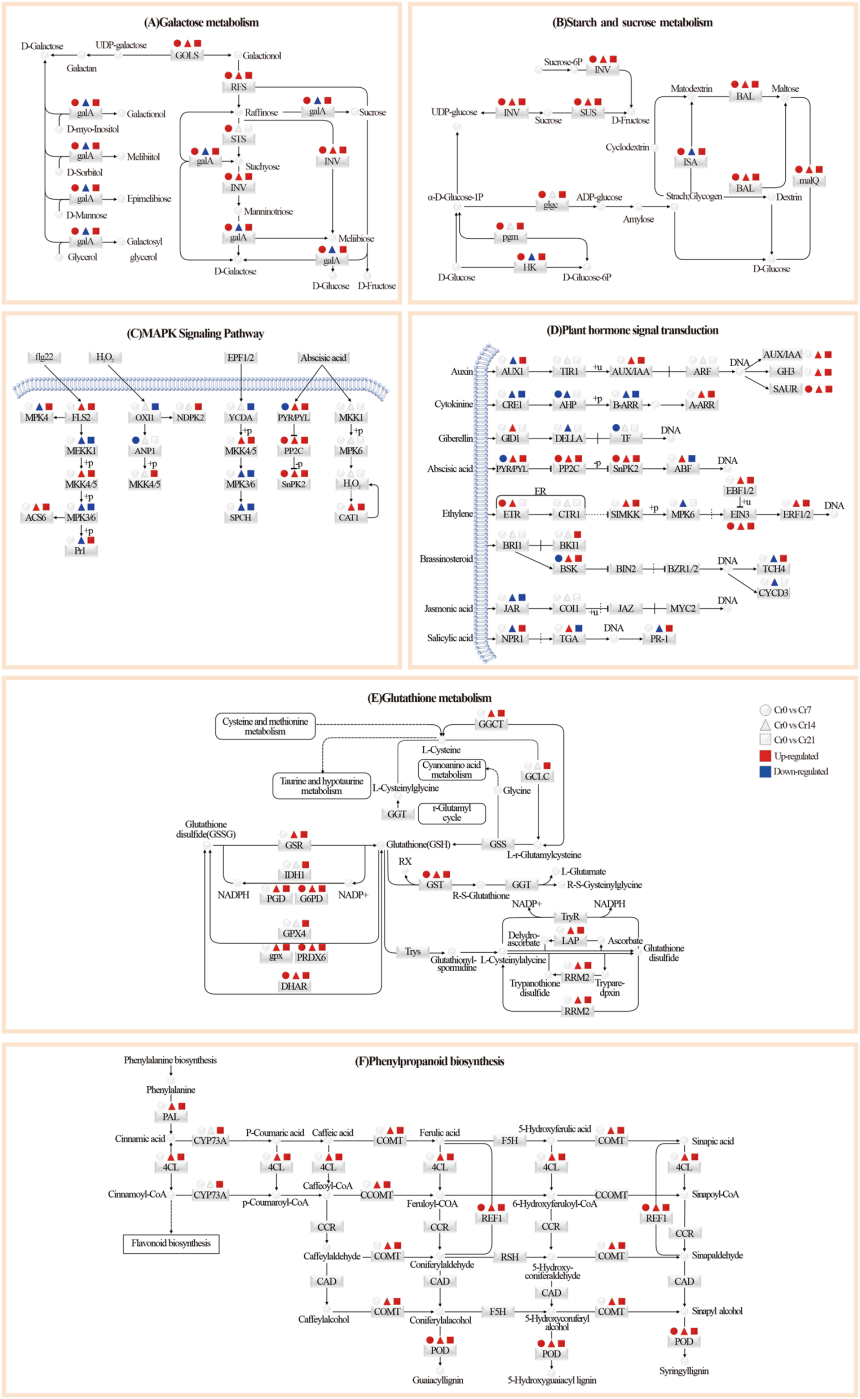
Changes of main metabolic pathways in *C. indica* after Cr stress. Red and blue indicate up-regulation and down-regulation, respectively, while gray indicates no significant change. See https://www.genome.jp/kegg/ for detailed annotations. Shanghai Majorbio Bio-pharm Technology platform provides all KEGG materials, and the copyright license for image use is obtained.

In Cr stressed environment, plant roots can jointly resist heavy metal poisoning by changing the content of their metabolites and gene expression of critical metabolic pathways^43^. This study revealed the molecular response mechanism of *C. indica* under Cr stress under time series through untargeted metabonomics studies combined with transcriptomic analysis. The results showed that DEGs involved in galactose, starch, and sucrose metabolism were significantly up-regulated in *C. indica* under short-term Cr stress, leading to a significant increase in carbohydrate content in *C. indica* (Figs. 3B, 8A,B). Many studies have shown that the upregulation of endoglucanase (EGLC) and β-amylase (SUS) in the starch and sucrose metabolism pathway will increase soluble sugar content in plants^41^. This phenomenon is essential in plant growth, development, and signal transduction in response to Cr stress^44^. In addition, galactose metabolism is an essential intermediate process of the carbohydrate cycle in plants, providing precursors for glucose metabolism and energy support for metabolic processes under stress, thus helping plants maintain nutritional balance in harsh environments^45^. Therefore, in the early stage of Cr stress, the rich carbohydrate metabolism process in *C. indica* may help to enhance plant tolerance to Cr for a short period.

In this study, in the middle stage of Cr stress, it was also found that plant hormone signal transduction and MAPK signaling pathway were significantly enhanced in *C. indica* (Fig. 8C,D). Generally speaking, plant hormones are vital regulatory factors mediating stress response, which can rapidly activate stress response mechanisms in various organelles, thus reducing oxidative damage in plants^41,46^. In order to induce the expression of various transport factors and the production of the Cr(VI) detoxification peptide chain, Enzymes involved in metabolic pathways in plants are activated in response to stress signals21. Heavy metals promote the expression of TFs and stress-responsive genes in plants and activate various signaling pathways, including MAPK signaling and hormone signaling^47^. In existing studies, Kumar et al. (2018) used *Arabidopsis thaliana* to reveal the activation pathway of MAPK signaling pathway in plants under heavy metal stress, mainly including ROS accumulation and changes in the antioxidant system. This study found that flg22 and Abscisic acid-related genes in the MAPK signaling pathway were significantly up-regulated in *C. indica* after the exogenous addition of Cr(VI) at the middle stage of stress. In auxin signal transduction, upregulation of ARF, which is a hub gene, in a high-Cr environment not only regulates the upregulation of downstream SAUR and GH3 to cope with adverse environmental conditions but also regulates the direction of plant growth factors by binding to Aux/IAA repressor proteins (Fig. 8C). This indicates that under Cr stress, with the extension of time, the growth strategy of *C. indica* changes, and the rapid root growth is conducive to plants' absorption of soil nutrients, thus conducive to the survival of plants in the stressed environment. Studies have shown that ABA is closely related to the signal transduction of plant stress resistance^48^. The results showed that in the middle stage of Cr stress, ABA receptor PYR/PYL-related genes were up-regulated and inhibited downstream PP2C and SnPK2-related genes, thus activating ABF binding factors and enhancing the ABA signal transduction process. In the JA signaling pathway, Verma et al. (2020) used transcriptomics to reveal the signal regulation of MYC2 in Arabidopsis thaliana, and the results showed that MYC2 mainly mediated the biosynthesis of proline. Meanwhile, in the salicylic acid signaling pathway, the results of^13^ showed that the NPR1 protein involved in REDOX regulation was mainly mediated by salicylic acid. Therefore, we speculate that IAA, ABA, jasmonic acid, and salicylic acid play a crucial role in enhancing plant Cr tolerance.

The plant can regulate heavy metals' transport through cell wall fixation, metal chelation, and vacuolar compartments. Among them, the absorption of heavy metals is mainly concentrated in the plant root cell wall, which can prevent external pollutants from entering the cell interior^18^. *Solanum nigrum L*.^43^, *Celosia argentea Linn*^45^, and *Sedum alfredii*, Studies on the distribution of heavy metals that the root cell wall is the leading site for binding heavy metals and plays a crucial role in plant response to Cr stress. The present study showed that after the exogenous addition of Cr(VI), DEGs involved in the regulation of phenylpropanoid biosynthesis were significantly up-regulated at the later stage of stress with the extension of stress time (Figs. 5H,I; 8E). Therefore, we speculated that *C. indica*, under long-term Cr stress, may activate the phenylpropanoid biosynthesis pathway in plants due to the increased accumulation of HMC in the roots, affecting the synthesis of coumarin and lignans, thus reducing the mobility and bioavailability of Cr. This can enhance the tolerance of *C. indica* to HMC^49,50^. Wang et al.^17^ also showed in recent studies that plants under heavy metal stress could enhance their binding ability with heavy metals by inducing their cell wall metabolism and reshaping their structure to enhance their tolerance to heavy metals. In addition, plants can jointly regulate the accumulation and transport of heavy metals by increasing cellulose and pectin contents and xylem cell wall thickness to alleviate heavy metals' toxic effects on plants^51^. Phenylpropanes (Fig. S1) and amino acid (Phenylalanine) contents (Fig. 3C,D) and the Phenylalanine metabolism pathway (Fig. 4C,D) were significantly up-regulated in late Cr stress. Among them, phenylalanine is catalyzed and oxidized to tyrosine by phenylalanine hydroxylase, which, together with tyrosine, synthesizes important hormone substances and participates in glucose metabolism and lipid metabolism^52^. Moreover, it can be converted into phenylpropane metabolites in secondary metabolic biosynthesis, including lignin and flavonoids, which greatly enhance the tolerance of plants to heavy metals^18^. In addition, the enhancement of the Phenylalanine metabolism pathway is consistent with transcriptome results, suggesting that cell wall metabolism-related pathways of *C. indica* may play a crucial role in plant response to heavy metal stress under prolonged heavy metal stress^13^. Phenylpropanes (Fig. S1) and amino acid (Phenylalanine) contents (Fig. 3C,D) and the Phenylalanine metabolism pathway (Fig. 4C,D) were significantly up-regulated in late Cr stress. Among them, phenylalanine is catalyzed and oxidized to tyrosine by phenylalanine hydroxylase, which, together with tyrosine, synthesizes important hormone substances and participates in glucose metabolism and lipid metabolism^52^. Moreover, it can be converted into phenylpropane metabolites in secondary metabolic biosynthesis, including lignin and flavonoids, which greatly enhance the tolerance of plants to heavy metals^18^. In addition, the enhancement of the Phenylalanine metabolism pathway is consistent with transcriptome results, suggesting that cell wall metabolism-related pathways of *C. indica* may play a crucial role in plant response to heavy metal stress under prolonged heavy metal stress^18^.

GSH is a plant antioxidant, mainly in the form of reduced glutathione (GSH) and oxidized glutathione (GSSG) in plants. It is involved primarily in the removal of ROS in plants and the chelation of heavy metals and plays an essential role in the stress resistance of plant cells^45^. This study showed that DEGs regulating GSH metabolism in *C. indica* roots were significantly up-regulated under two treatment times of Cr14 and Cr21 (Figs. 5H,I; 8F). This may be because plants exposed to heavy metal stress for a long time can produce a large amount of ROS. However, excessive ROS accumulation will eventually lead to lipid peroxidation of the cell membrane and damage the function of the cell membrane. Therefore, genes that regulate GSH metabolism in plants are induced to be expressed in response to oxidative damage caused by stress^36^. However, some studies have shown that ROS in plants can be independently produced in different compartments and serve stress-sensing and signaling purposes in plants to regulate gene expression and induce stress recovery^46^. It is worth noting that GSH can not only capture and bind heavy metal ions attached to the enzyme protein sulfhydryl but also reduce them too acidic substances through the combination of sulfhydryl with free radicals in plants to accelerate the removal of free radicals, thus enhancing the tolerance of plants to heavy metals^53^. Meanwhile, Yu et al.^45^ revealed the detoxification mechanism of GSH in plants of *Celosia argentea Linn* under heavy metal stress through multi-omics analysis. The results showed that GSH is a precursor to phytochelatin peptides (PCs) using a synthase to complex free HM ions in plant cells. This HM complex is then delivered to plant vacuoles via tonoplast membrane transporters for eventual detoxification. Hasanuzzaman et al.^54^ reported that plants' antioxidant content would increase significantly under oxidative stress, dramatically enhancing plants' stress resistance. In addition, WGCNA showed that the central gene of the MEdarkolivegreen module was significantly positively correlated with the GSH content in plants (P < 0.001), and its expression was significantly increased in the Cr21 group (Fig. 6H,I). This is similar to the gene expression results of the GSH metabolic pathway in Morus alba L. and Pepper^36,55^. Essential functional genes involved in glutathione S-transferase and Glutathione metabolism were significantly up-regulated under heavy metal stress, greatly enhancing plant stress resistance.

This study found that terpenoid content in plant bodies increased significantly under heavy metal stress, mainly including Ginkgolide C, 17-Oxogrindelic acid, Limonin, and Gibberellin A44 (Table S1). This may be because terpenoid metabolites can significantly scavenge hydroxyl radicals and superoxide anions in plants, reduce the level of malondialdehyde in cells, and enhance the activities of superoxide dismutase, catalase, and glutathione peroxidase, reflecting vigorous antioxidant activity. To alleviate oxidative damage from heavy metals^41,56^. At the same time, triterpenoids are an essential class of natural antioxidant active substances. Studies have shown that triterpenoid metabolites play a crucial role in complex biochemical processes such as stress response and cell physiological reaction in plants, with scavenging solid ability of 1, 1-diphenyl-2-trinitrophenylhydrazide (DPPH) free radicals, and the scavenging rate is as high as 90%. Moreover, it positively correlates with concentration^13^. The contents of amino acids (L-Glutamate, Glutamine, and Glycine) and flavonoid metabolites at the late stage of Cr stress (Fig. 3D; Fig. S1) and glutamate metabolism (Fig. 4D) were significantly up-regulated. In plants, amino acids can effectively chelate metal ions in the cytoplasm to reduce the toxic effect of heavy metals on plants^56^. L-Glutamate is an intermediate for oxidized amino acids, which plants can use for secondary metabolisms, such as the biosynthesis of glutamine, proline, and lysine^57^. Glutamine is an essential precursor of glutathione biosynthesis in plants. Glycine is an antioxidant involved in heavy metal chelation in plants, and the presence of these metabolites plays a crucial role in plant survival and stress resistance^58^. In addition, proline is an ideal plant osmotic regulator, a protective substance for enzymes in cell membranes and plants, and a free radical scavenger^41^ which can protect the growth of *C. indica* under heavy metal stress. Mwamba et al. (2022) revealed the adaptive mechanism of *Brassicanapus* to heavy metal stress through metabonomics analysis. The results showed that phenolic compounds were involved in response to heavy metal stress, among which flavonoids were significantly induced. They indicated that the antioxidant mechanism was the final response strategy to heavy metal stress.

In addition, metabonomics analysis showed that the content of plant secondary metabolites, flavonoids, was significantly increased, effectively alleviating the toxic effects of Cr stress. Flavonoids are important secondary metabolites. The hydrogen atom on the phenolic hydroxyl group of flavonoids can combine with peroxyl radicals to form flavonoid radicals, thereby ending the free radical chain reaction and enhancing the ability of plants to resist environmental stresses^8^. Previous studies have shown that as defensive antioxidants, flavonoids (chalcone, naringenin, eriodictyol, dihydroquercetin) have substantial free radical scavenging ability and antioxidant activity and can block free radical chain reaction in the form of free radical receptors. At the same time, it can form chelating substances with heavy metal ions, thereby slowing down oxidative damage^57^. Moreover, flavonoids can also interact with membrane phospholipids in plant cell membranes, preventing toxic small molecules from entering the hydrophobic region of the phospholipid bilayer and thereby protecting the integrity of plant cell membranes^34,35^. In this study, it was found that the contents of most classified flavonoids (Coumestrol, Quercetin 3-beta-D-glucoside, Apigenin 7-O-glucoside, Diosmetin and (+ /−) -catechin) increased with the time of Cr stress (Table S1). These results indicated that under heavy metal stress, plants might activate their biosynthetic pathway of flavonoids to produce a large number of reducing metabolic substances, remove a large number of ROS induced by heavy metal stress in plants, alleviate oxidative damage in plants, and reduce membrane lipid peroxidation. Thus, it can enhance plants' tolerance to heavy metal stress^20^. In conclusion, *C. indica* can resist Cr stress mainly through carbohydrate metabolism (galactose, starch, and sucrose metabolism) in the early stage of Cr stress (Cr7). With the extension of Cr stress time, in the middle stage of stress (Cr14), plants mainly use plant hormone signal transduction and MAPK signaling pathway to regulate their stress response system to cope with Cr stress jointly. In anaphase of stress (Cr21), *C. indica* mainly activates its Glutathione metabolism and Phenylpropanoid The expression of genes related to biosynthesis and the accumulation of metabolites (phenylpropanoids, phenylalanine, flavonoids, and L-Glutamate) can jointly resist the toxicity of Cr through endogenous and exogenous molecular response defense mechanisms, thus enhancing the tolerance of plants to Cr stress.

## Conclusions

This study showed that *C. indica* showed corresponding changes in plant growth and physiological metabolite levels under Cr stress at different exposure times. Due to the toxicity of Cr itself, plant growth was significantly inhibited, the biosynthesis of photosynthetic pigments was damaged, and the expression levels of enzymes were promoted (POD, SOD, and APX). Non-enzymes-promoted antioxidants in plants were reduced considerably. In addition, this study revealed the molecular response mechanism of *C. indica* under different stress times through transcriptome and metabolomics analysis. The results showed that *C. indica* maintained its energy supply through galactose, starch, and sucrose metabolism in the early stages of stress (Cr7), it enhanced the stress resistance of *C. indica* under heavy metal stress. With the extension of stress time, the plant hormone signal transduction and MAPK signaling pathway processes of *C. indica* during the middle stage of stress (Cr14) are affected, thus altering the plant growth strategy. Finally, in the later stages of stress (Cr21), *C. indica* mainly activates the expression of Glutathione metabolism and Phenylpropanoid biosynthesis genes and the accumulation of antioxidant substances (phenylalanine, flavonoids, and L-Glutamate). Both endogenous and exogenous defense mechanisms can jointly resist Cr poisoning, enhancing the tolerance of *C. indica* to Cr. The results of this study are expected to provide new insights into the growth, physiological, and molecular response mechanisms of *C. indica* under Cr stress.

### Supplementary Information

Supplementary Tables.

Supplementary Figures.

## Data Availability

Sequence data that support the findings of this study have been deposited in the National Center for Biotechnology Information with the primary accession code PRJNA1040463, https://www.ncbi.nlm.nih.gov/bioproject/PRJNA1040463/.
