# Supplementary material for: Integrated transcriptome and metabolomics analyses revealed key functional genes in Canna indica under Cr stress
Source: Sci Rep. 2024 Jun 18;14:14090. doi: 10.1038/s41598-024-64877-w (PMC11189463; doi:10.1038/s41598-024-64877-w)
Supplement: Supplementary file 2 — Supplementary Figures. [file 41598_2024_64877_MOESM2_ESM.pdf]

Fig. S1 Classification of differentially expressed metabolites in the roots of C. indica under Cr stress. (A) secondary metabolites and (B) lipids.

Fig. S2 Changes in differential metabolites between comparison groups. (A) Volcano plots of up-regulated and down-regulated DEMs, the size of the bubbles indicates the amount of differentially expressed metabolites. (B) The top 10 signs of the up-regulated and down-regulated DEMs, the length of the column represents the amount of differential metabolites.

Fig. S3 Detailed annotation information of transcripts in nonredundant (NR), EuKaryotic Orthologous Groups (KOG), KEGG, and GO databases.

Fig. S4 (A) Correlation analysis of gene expression patterns in control and Cd-treated groups. (B) Principal component analysis (PCA) of FPKM profiles in control and Cd-treated groups.

Fig. S1

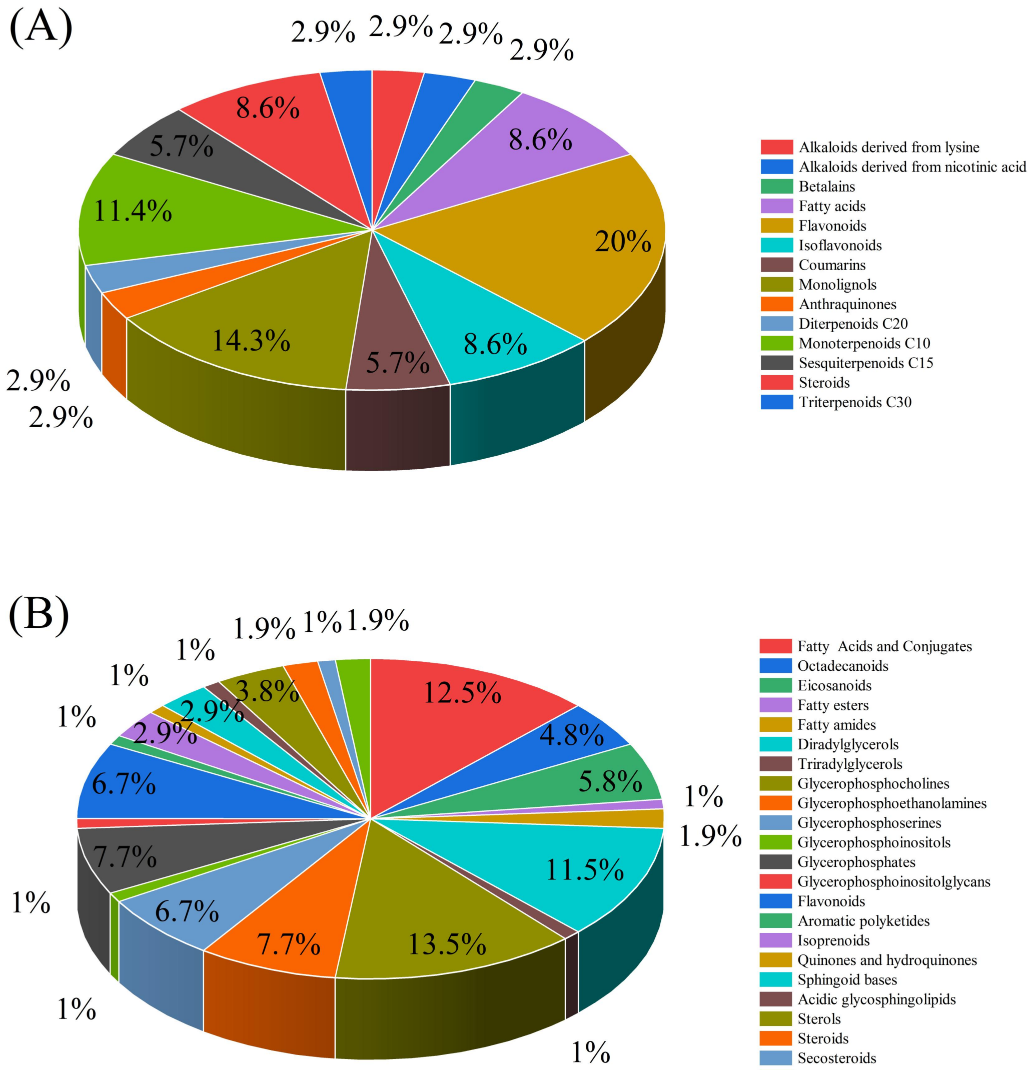
(A)
2.9%
2.9%
2.9%
2.9%
8.6%
5.7%
8.6%
11.4%
20%
14.3%
5.7%
8.6%
2.9%
2.9%
Alkaloids derived from lysine
Alkaloids derived from nicotinic acid
Betalains
Fatty acids
Flavonoids
Isoflavonoids
Coumarins
Monolignols
Anthraquinones
Diterpenoids C20
Monoterpenoids C10
Sesquiterpenoids C15
Steroids
Triterpenoids C30
(B)
1.9%
1%
1.9%
1%
1%
1%
12.5%
3.8%
2.9%
2.9%
4.8%
6.7%
5.8%
1%
1.9%
7.7%
11.5%
1%
6.7%
7.7%
13.5%
1%
1%
Fatty Acids and Conjugates
Octadecanoids
Eicosanoids
Fatty esters
Fatty amides
Diradylglycerols
Triradylglycerols
Glycerophosphocholines
Glycerophosphoethanolamines
Glycerophosphoserines
Glycerophosphoinositols
Glycerophosphates
Glycerophosphoinositolglycans
Flavonoids
Aromatic polyketides
Isoprenoids
Quinones and hydroquinones
Sphingoid bases
Acidic glycosphingolipids
Sterols
Steroids
Secosteroids
Steroid conjugates

Fig. S2

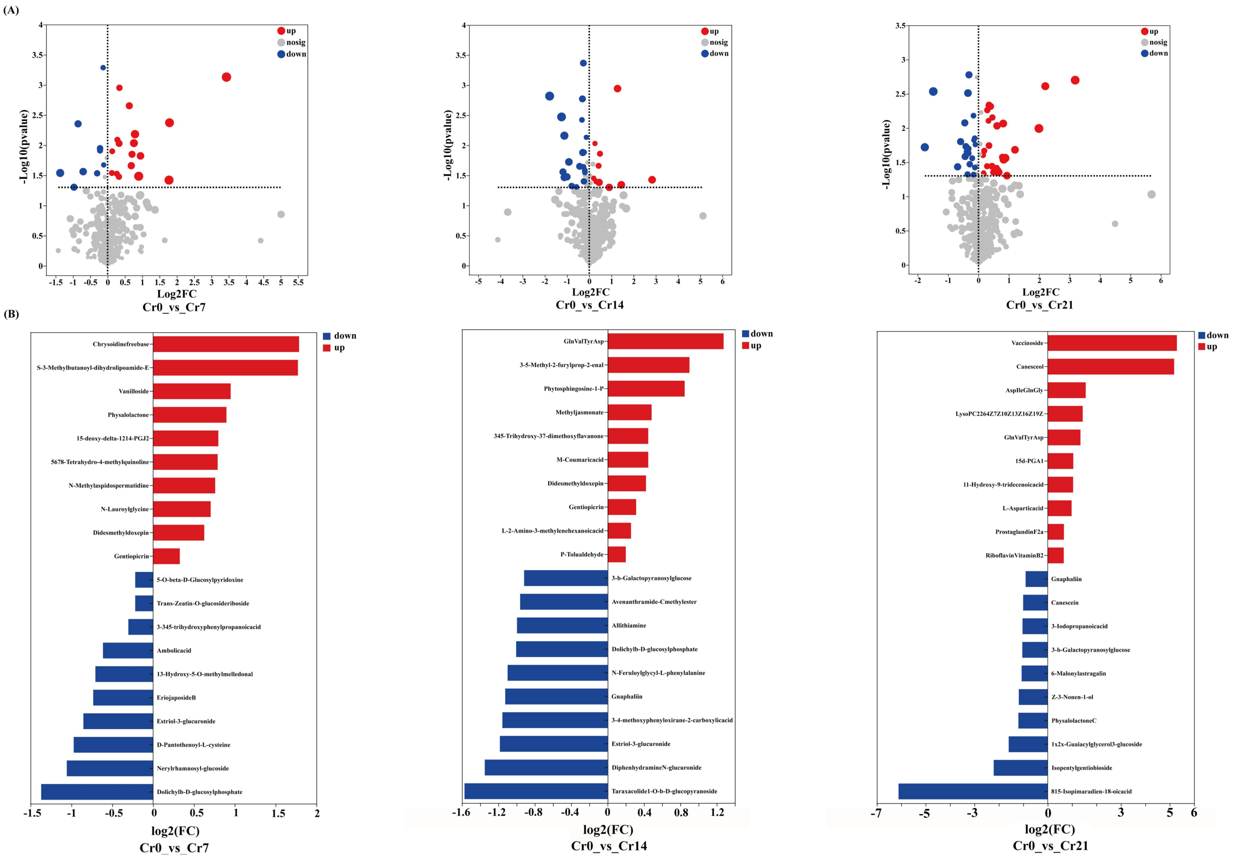
(A)
up
nosig
down
-Log10(pvalue)
Log2FC
Cr0_vs_Cr7
Cr0_vs_Cr14
Cr0_vs_Cr21
(B)
down
up
Chrysoidinefreebase
S-3-Methylbutanoyl-dihydrolipoamide-E
Vanilloside
Physalolactone
15-deoxy-delta-1214-PGJ2
5678-Tetrahydro-4-methylquinoline
N-Methylaspidospermatidine
N-Lauroylglycine
Didesmethyldoxepin
Gentiopicrin
5-O-beta-D-Glucosylpyridoxine
Trans-Zeatin-O-glucosideriboside
3-345-trihydroxyphenylpropanoicacid
Ambolicacid
13-Hydroxy-5-O-methylmelledonal
EriojaposideB
Estriol-3-glucuronide
D-Pantothenoyl-L-cysteine
Nerylrhamnosyl-glucoside
Dolichylb-D-glucosylphosphate
log2(FC)
Cr0_vs_Cr7
GlnValTyrAsp
3-5-Methyl-2-furylprop-2-enal
Phytosphingosine-1-P
Methyljasmonate
345-Trihydroxy-37-dimethoxyflavanone
M-Coumaricacid
Didesmethyldoxepin
Gentiopicrin
L-2-Amino-3-methylenehexanoicacid
P-Tolualdehyde
3-b-Galactopyranosylglucose
Avenanthramide-Cmethylester
Allithiamine
Dolichylb-D-glucosylphosphate
N-Feruloylglycyl-L-phenylalanine
Gnaphaliin
3-4-methoxyphenyloxirane-2-carboxylicacid
Estriol-3-glucuronide
DiphenhydramineN-glucuronide
Taraxacolide1-O-b-D-glucopyranoside
log2(FC)
Cr0_vs_Cr14
Vaccinoside
Canesceol
AspIleGlnGly
LysoPC2264Z7Z10Z13Z16Z19Z
GlnValTyrAsp
15d-PGA1
11-Hydroxy-9-tridecenoicacid
L-Asparticacid
ProstaglandinF2a
RiboflavinVitaminB2
Gnaphaliin
Canescein
3-Iodopropanoicacid
3-b-Galactopyranosylglucose
6-Malonylastragalin
Z-3-Nonen-1-ol
PhysalolactoneC
1x2x-Guaiacylglycerol3-glucoside
Isopentylgentiobioside
815-Isopimaradien-18-oicacid
log2(FC)
Cr0_vs_Cr21

Fig. S3

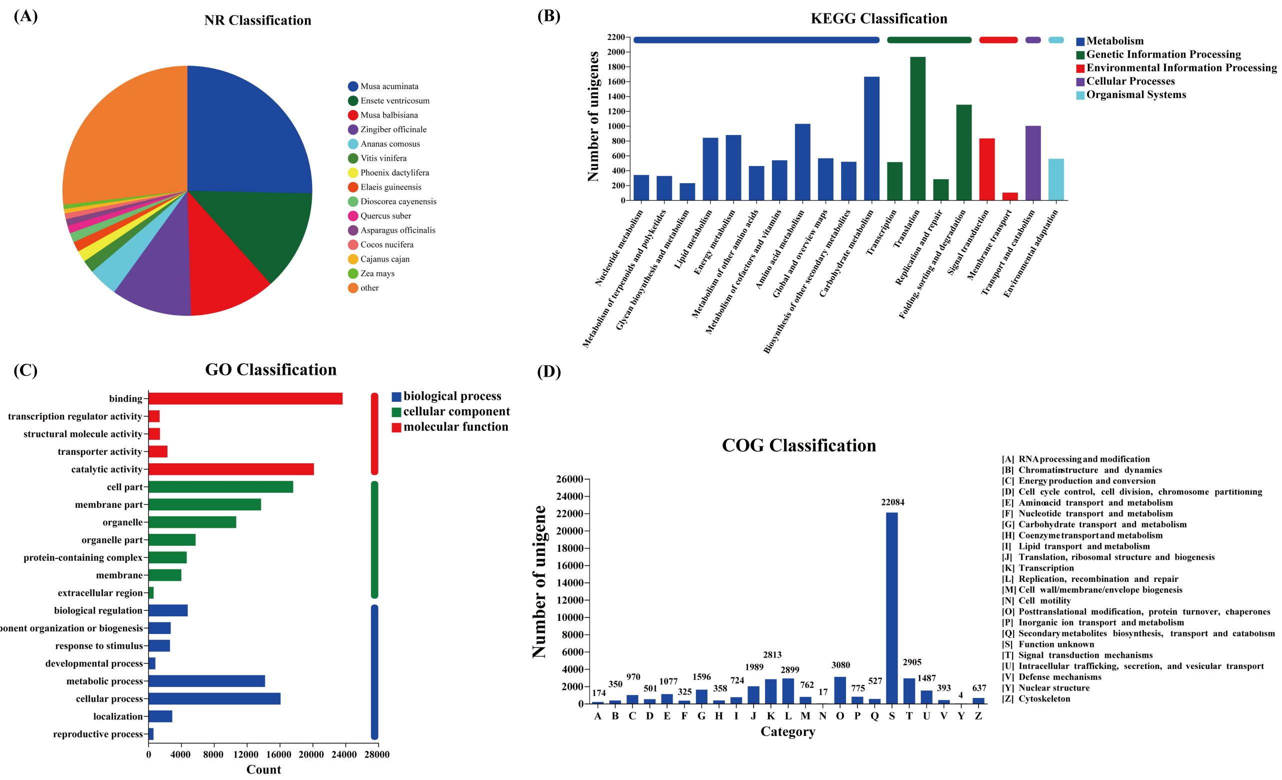
(A)
NR Classification
Musa acuminata
Ensete ventricosum
Musa balbisiana
Zingiber officinale
Ananas comosus
Vitis vinifera
Phoenix dactylifera
Elaeis guineensis
Dioscorea cayenensis
Quercus suber
Asparagus officinalis
Cocos nucifera
Cajanus cajan
Zea mays
other
(B)
KEGG Classification
Metabolism
Genetic Information Processing
Environmental Information Processing
Cellular Processes
Organismal Systems
Number of unigenes
0
200
400
600
800
1000
1200
1400
1600
1800
2000
2200
Nucleotide metabolism
Metabolism of terpenoids and polyketides
Glycan biosynthesis and metabolism
Lipid metabolism
Energy metabolism
Metabolism of other amino acids
Metabolism of cofactors and vitamins
Amino acid metabolism
Global and overview maps
Biosynthesis of other secondary metabolites
Carbohydrate metabolism
Transcription
Translation
Replication and repair
Folding, sorting and degradation
Signal transduction
Membrane transport
Transport and catabolism
Environmental adaptation
(C)
GO Classification
biological process
cellular component
molecular function
binding
transcription regulator activity
structural molecule activity
transporter activity
catalytic activity
cell part
membrane part
organelle
organelle part
protein-containing complex
membrane
extracellular region
biological regulation
cellular component organization or biogenesis
response to stimulus
developmental process
metabolic process
cellular process
localization
reproductive process
0
4000
8000
12000
16000
20000
24000
28000
Count
(D)
COG Classification
Number of unigene
0
2000
4000
6000
8000
10000
12000
14000
16000
18000
20000
22000
24000
26000
174
350
970
501
1077
325
1596
358
724
1989
2813
2899
762
17
3080
775
527
22084
2905
1487
393
4
637
A B C D E F G H I J K L M N O P Q S T U V Y Z
Category
[A] RNA processing and modification
[B] Chromatin structure and dynamics
[C] Energy production and conversion
[D] Cell cycle control, cell division, chromosome partitioning
[E] Amino acid transport and metabolism
[F] Nucleotide transport and metabolism
[G] Carbohydrate transport and metabolism
[H] Coenzyme transport and metabolism
[I] Lipid transport and metabolism
[J] Translation, ribosomal structure and biogenesis
[K] Transcription
[L] Replication, recombination and repair
[M] Cell wall/membrane/envelope biogenesis
[N] Cell motility
[O] Posttranslational modification, protein turnover, chaperones
[P] Inorganic ion transport and metabolism
[Q] Secondary metabolites biosynthesis, transport and catabolism
[S] Function unknown
[T] Signal transduction mechanisms
[U] Intracellular trafficking, secretion, and vesicular transport
[V] Defense mechanisms
[Y] Nuclear structure
[Z] Cytoskeleton

Fig. S4

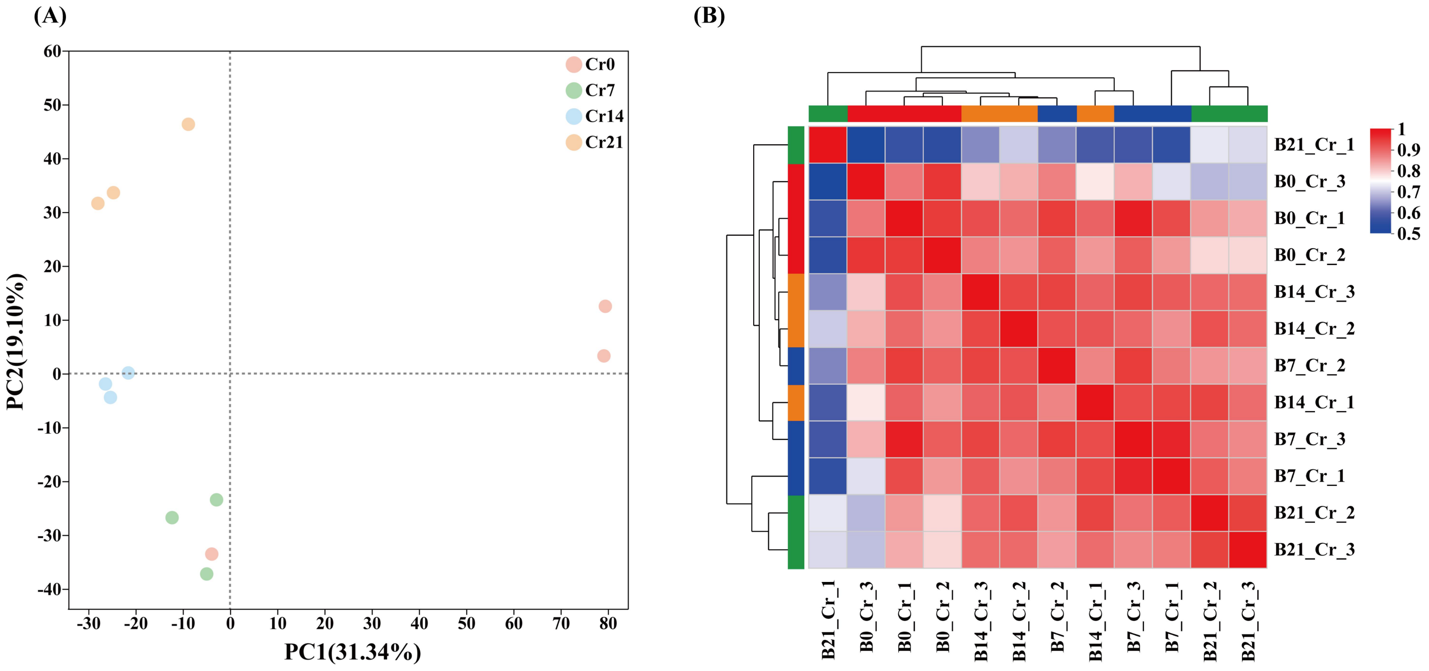
(A)
(B)
PC1(31.34%)
PC2(19.10%)
Cr0
Cr7
Cr14
Cr21
B21_Cr_1
B0_Cr_3
B0_Cr_1
B0_Cr_2
B14_Cr_3
B14_Cr_2
B7_Cr_2
B14_Cr_1
B7_Cr_3
B7_Cr_1
B21_Cr_2
B21_Cr_3
1
0.9
0.8
0.7
0.6
0.5
